# Relationship between sleep disorders and the efficacy of immune checkpoint inhibitors in older patients with non-small cell lung cancer

**DOI:** 10.3389/fonc.2025.1713801

**Published:** 2026-01-09

**Authors:** Zheng Liu, Hongjun Zhu, Kan Wang

**Affiliations:** Department of Thoracic Surgery, The First People’s Hospital of Shangqiu, Shangqiu, Henan, China

**Keywords:** efficacy, immune checkpoint inhibitor, non-small cell lung cancer, older patients, sleep disorders

## Abstract

**Background:**

Immune checkpoint inhibitors (ICIs) represent a critical treatment modality for older adults with advanced non-small cell lung cancer (NSCLC). The objective of this study was to investigate the potential relationship between sleep disorders, a condition commonly observed in this older population, and the efficacy of ICIs in treating advanced NSCLC.

**Methods:**

A total of 495 older patients with NSCLC who had distant metastases or local infiltration and were ineligible for surgical resection were consecutively enrolled in the study. All patients received either ICI monotherapy or combination therapy, and were followed up for 24 months. Sleep quality was assessed four times using the Pittsburgh Sleep Quality Index to assess the average severity of sleep disorders. Treatment conditions, clinical outcomes, and Grade 3–4 adverse events were documented, and associations were evaluated using multivariate logistic regression or Cox regression analysis.

**Results:**

Sleep disorders were associated with a reduced likelihood of achieving complete response/partial response/stable disease for ICI efficacy at 3 months (P = 0.002), an increased risk of gastrointestinal, liver, and lung toxicity over the 24-month follow-up (P = 0.014, P = 0.012, P = 0.036), and a decreased probability of survival at 24 months (P = 0.004). Sleep disorders were also associated with reduced progression-free survival and overall survival during the 24 months (P = 0.048, P = 0.002).

**Conclusion:**

Among older NSCLC patients receiving ICI treatment, sleep disorders are significantly associated with poorer treatment efficacy and a higher risk of multisystem adverse reactions. The findings of this study provide preliminary evidence for integrating sleep assessment into individualized ICI treatment strategies in this population.

## 1 Introduction

According to the Global Cancer Statistics 2022, approximately 2.5 million new lung cancer cases were diagnosed globally, and 1.8 million deaths were attributed to the disease (1). Consequently, lung cancer ranks first in both incidence and mortality rates across all cancer types. Notably, non-small cell lung cancer (NSCLC), which accounts for around 85% of lung cancer cases, is the most prevalent subtype among lung malignancies (2).

It is well-established that the risk of developing NSCLC increases significantly with age. Specifically, individuals aged 60 and older are at higher risk, with the peak incidence occurring between 70 and 80 years of age (3). For older adults, who may experience gradual declines in physical function and often have multiple comorbidities, the diagnosis and treatment of NSCLC present unique challenges.

Statistical data indicate that approximately 50%-70% of older adults with NSCLC are diagnosed at an advanced stage (4). At this stage, the cancer has often metastasized extensively or tumors cannot be surgically resected due to severe local invasion. As a result, the main treatment options for these patients are palliative therapies such as radiotherapy and chemotherapy, leading to generally poor prognoses.

Immune checkpoint inhibitors (ICIs) are a class of anticancer drugs that block immune checkpoint proteins, lift the suppression of the immune system by tumors, and activate the body’s immune cells to recognize and eliminate tumor cells (5). Their introduction has offered new treatment alternatives for patients with widely metastatic and unresectable NSCLC. Indeed, multiple studies have demonstrated that ICIs, used either alone or in combination with other treatments, can significantly prolong the survival of NSCLC patients (6, 7).

Over the past two to three years, a group of researchers has noted that sleep may be associated with the efficacy of ICIs. In 2022, a pilot study (n=32) was the first to identify a significant association between sleep latency >15 minutes and a diagnosis of metastatic cancer among cancer patients receiving ICI treatment (8). While this finding did not directly address ICI efficacy, it provided preliminary clues for subsequent research. A preliminary study conducted in 2023 (n=62) further validated at the mechanistic level that sleep disorders were correlated with elevated levels of soluble CTLA-4 (a negative correlate of ICI efficacy) and the inflammatory cytokine IL-6. This suggests that sleep may influence ICI therapeutic responses indirectly by regulating immuno-inflammatory pathways (9). Most recently, a 2024 prospective study involving 171 patients with advanced NSCLC directly reported that sleep disorders may serve as an independent predictor of shortened progression-free survival (PFS) following ICI treatment. It was also associated with lower objective response rates and disease control rates (10). The sample sizes of the aforementioned studies are generally small, and only one study was specifically conducted on patients with NSCLC. The current evidence is relatively limited and insufficient to support a definitive conclusion regarding the association between sleep disorders and the efficacy of ICIs. Therefore, additional studies with larger sample sizes and stronger target specificity are still needed to further validate and refine conclusions in this field.

Our study was conducted almost concurrently with the aforementioned studies. We enrolled hundreds of older patients with inoperable advanced NSCLC prospectively, aiming to explore the potential relationship between sleep disorders and ICI efficacy in this specific population. The findings of this study will further enrich the evidence base for evidence-based medicine in this field and provide clinical evidence to guide ICI treatment for older patients with advanced NSCLC.

## 2 Materials and methods

### 2.1 Ethical requirements

This study was conducted in strict accordance with the World Medical Association’s Declaration of Helsinki (11). All participants and their families provided informed consent for study participation and signed the corresponding consent forms.

### 2.2 Subjects

Older patients with NSCLC who received treatment at the Department of Thoracic Surgery, First People’s Hospital of Shangqiu, between October 1, 2019, and December 31, 2022, were consecutively enrolled.

The inclusion criteria were as follows: (1) Participants aged 60 years or older. (2) Pathologically confirmed NSCLC diagnosis. (3) Patients had no prior history of antitumor treatment. (4) According to multidisciplinary consultations from the hospital’s Department of Thoracic Surgery and Oncology, patients had advanced disease (stage IIIB, IIIC, or IV) due to extensive metastasis or local infiltration, rendering them ineligible for surgical resection, and were planned to receive ICI treatment. (5) From baseline, patients received ICI treatment for at least 3 months. This is because an excessively short ICI treatment course may lead to biases in the assessment of ICI efficacy. Treatment strategies could include ICI monotherapy, or ICI combined with radiotherapy and/or chemotherapy. (6) All patients and their family members consented to cooperate in completing a 24-month follow-up period, unless the patient died. (7) Normal cognitive and communication abilities, enabling cooperation with the study procedures. (8) No history of other malignancies, stable chronic disease management, and no significant health risks precluding study participation.

The exclusion criteria were as follows: (1) Patients not meeting the above inclusion criteria were excluded at baseline. (2) During the follow-up period, there were significant changes in the patients themselves, their families, or their living environments, except for conditions related to NSCLC, which may interfere with sleep during this period. (3) During the follow-up period, exacerbation of pre-existing chronic diseases or the development of new severe illnesses increased the risk of the patients continuing to participate in this study. (4) During the follow-up period, death due to causes other than NSCLC. (5) Loss to follow-up occurred during the follow-up period.

### 2.3 Baseline data collection

Baseline data were collected through medical record review and face-to-face interviews, including: (1) Demographic data. (2) History of chronic diseases. (3) Risk factors for NSCLC. (4) Nutritional data. (5) Peripheral immune and inflammatory data. (6) Characteristics of NSCLC (including pathological data).

Patients’ chronic disease histories were confirmed based on diagnostic reports and treatment records. Risk factors for NSCLC were determined in accordance with *a priori* definitions, as detailed in **Supplementary Table 1**. All serological tests were routine clinical items performed in the hospital’s laboratory, with quality control overseen by the laboratory’s quality director. Pathological data for NSCLC were obtained from pathology reports.

### 2.4 Definition of the follow-up

All patients underwent a 24-month follow-up. Baseline was defined as the time point when ICI treatment was initiated. The end event of follow-up was either the last day of the 24-month period or death due to NSCLC during the follow-up period.

### 2.5 Sleep assessment and grouping

Each patient was assessed using the Pittsburgh Sleep Quality Index (PSQI) questionnaire on four separate occasions: at baseline, and at the end of the 1st, 2nd, and 3rd months of follow-up. These four assessments were sequentially defined as the first, second, third, and fourth assessments. The average value of the four results was used for data analysis.

The PSQI is a widely used standardized questionnaire for assessing sleep quality, consisting of 19 self-rated items and 5 investigator-rated items. All items are ultimately categorized into 7 dimensions: daytime dysfunction, sleep medications, sleep disturbances, sleep latency, sleep duration, sleep efficiency, and subjective sleep quality. Each dimension is scored on a 0–3 scale (0 = no problem, 3 = severe problem). The total score is the sum of the scores of the 7 dimensions, ranging from 0 to 21, and serves as the core indicator for judging sleep quality. A higher total score indicates poorer sleep quality. The commonly used clinical interpretation criteria are as follows: 0–5 points indicate good sleep quality; 6–10 points indicate moderate sleep quality; 11–15 points indicate poor sleep quality; and 16–21 points indicate extremely poor sleep quality (12, 13).

In this study, a PSQI total score greater than 5 was defined as indicating sleep disorders, and these patients were assigned to the sleep disorder group; patients with a PSQI total score of ≤ 5 were assigned to the normal sleep group.

### 2.6 Treatment and outcomes

Each patient was followed up at 3 months, 6 months, 12 months, 18 months, and 24 months, respectively. The follow-up was conducted by reviewing medical records and telephone communication, during which the treatment regimen, characteristics of various treatments (ICIs, chemotherapy, radiotherapy), ICI efficacy, adverse reactions (Grade 3-4), PFS, and overall survival (OS) were documented.

Based on drug availability and evolving healthcare policies, patients received either Pembrolizumab or Nivolumab therapy for NSCLC (14). Pembrolizumab was administered intravenously at 200 mg every 3 weeks, while Nivolumab was given at 240 mg every 2 weeks. ICI treatment was discontinued under the following conditions: (1) Persistent and significant NSCLC progression, indicating treatment failure. (2) Unmanageable drug-related toxicity. (3) Continuous complete remission for 24 months.

Some patients also received concurrent radiotherapy and/or chemotherapy. Among these treatments, radiotherapy is exclusively palliative, with the core purpose of relieving local symptoms or controlling local tumor progression, and the dose is mainly ≤ 50 Gy; chemotherapy regimens are selected based on the patient’s performance status, liver and kidney function, and comorbidities, primarily using well-tolerated regimens commonly used in clinical practice for older NSCLC patients, including Pemetrexed plus Cisplatin, Paclitaxel plus Carboplatin, and Gemcitabine plus Cisplatin. The goal is to enhance antitumor efficacy through the synergistic effect of chemotherapy and ICIs, while also ensuring treatment safety.

During the first 6 months of ICI treatment, patients’ status was evaluated approximately every 6 weeks using thoracic CT scans and clinical assessments. From the 7th to the 12th month of ICI treatment, evaluations were performed approximately every 8 weeks. For treatment durations exceeding 12 months, assessments were conducted approximately every 12 weeks.

Based on the results of thoracic CT scans, treatment efficacy was classified into the four categories (15): (1) Complete response (CR): complete disappearance of all visible tumor lesions. (2) Partial response (PR): ≥30% reduction in the sum of the products of the longest diameter and perpendicular diameter of tumor lesions. (3) Stable disease (SD): failure to meet PR criteria, with ≤20% increase in tumor measurements. (4) Progressive disease (PD): >20% increase in tumor measurements or appearance of new lesions.

### 2.7 Statistical analysis

Continuous variables were reported as mean ± standard deviation, and inter-group differences were analyzed using independent samples t-tests. Categorical variables were presented as frequencies and percentages, with group comparisons performed via chi-square tests.

Multivariate logistic regression was used to assess the association of sleep disorders with ICI efficacy at 3 months, adverse reactions (Grade 3-4) during the follow up, and outcome at 24 months. Odds ratios (ORs), 95% confidence intervals (95%CIs) and P values were reported. In this multivariate model, demographic data, disease history, risk factors for NSCLC, nutritional data, peripheral immune and inflammatory data, tumor characteristics, treatment regimen, and ICI course were adjusted. When necessary, subgroup analysis and interaction tests were used to verify the impact of key confounding factors on the results of this study.

Multivariate Cox regression and Kaplan-Meier survival analysis were used to assess the association of sleep disorder with PFS and OS. In the multivariate Cox regression, the potential confounding factors mentioned above were adjusted, and the hazard ratios (HRs), 95%CIs and P values were reported.

Given the potential baseline data imbalance between the two groups, after completing the aforementioned multivariate regression analyses, propensity score matching was optionally employed for supplementary analysis. The matching variables were consistent with the baseline confounding factors included in the aforementioned multivariate regression model, aiming to reduce the potential impact of baseline confounding factors on the study results and enhance the reliability of the conclusions.

In all the analyses described above, a P value < 0.05 was indicative of a statistically significant difference or correlation (16). All the aforementioned analyses were performed using SPSS 27.0.

Additionally, GPowerWin_3.1.9.7 was used to conduct a *post-hoc* analysis to verify whether the sample size provided sufficient statistical power (17).

## 3 Results

### 3.1 Subjects

At baseline, 514 patients who met the eligibility criteria were enrolled in this study. During the follow-up period, 19 patients were excluded from the study due to loss to follow-up (n = 10), significant changes in nighttime sleep environment (n = 5), sudden acute myocardial infarction or cerebral infarction (n = 2), and accidental death from non-NSCLC causes (n = 2). Ultimately, 495 patients (accounting for 96.3%) were included in the final analysis of this study.

Based on the average results of four sleep assessments, 210 patients with a total PSQI score > 5 were assigned to the sleep disorder group, while the remaining 285 patients with a total PSQI score ≤ 5 were assigned to the normal sleep group. **Table 1** presents all PSQI-related scores of patients in the two groups. The results showed that compared with the normal sleep group, the sleep disorder group had significantly higher scores in terms of the total PSQI score at each assessment, the average of total scores across all assessments, and the average score of each PSQI dimension (all P < 0.001).

**Table 1:** Sleep status of older patients in the first 3 months of follow-up period.

Variable	Sleep disorder group	Normal sleep group	χ² or t value	P value
Total (n)	210 (100.0)	285 (100.0)	–	–
PSQI total score				
1st	10.6 ± 2.7	2.5 ± 1.5	42.924	<0.001
2nd	10.8 ± 3.0	2.6 ± 1.5	40.151	<0.001
3rd	9.8 ± 3.1	2.3 ± 1.4	36.452	<0.001
4th	10.8 ± 3.6	2.4 ± 1.4	35.994	<0.001
Average	10.5 ± 2.9	2.5 ± 1.4	40.705	<0.001
Each item average score				
Daytime dysfunction	1.51 ± 0.43	0.36 ± 0.22	38.589	<0.001
Sleep medications	1.50 ± 0.44	0.36 ± 0.24	37.418	<0.001
Sleep disturbances	1.51 ± 0.45	0.36 ± 0.23	37.269	<0.001
Sleep latency	1.50 ± 0.44	0.35 ± 0.23	37.955	<0.001
Sleep duration	1.49 ± 0.42	0.35 ± 0.23	38.923	<0.001
Sleep efficiency	1.36 ± 0.39	0.44 ± 0.26	31.177	<0.001
Subjective sleep quality	1.58 ± 0.51	0.22 ± 0.21	40.602	<0.001

PSQI = Pittsburgh Sleep Quality Index. Continuous variables are expressed as mean ± standard deviation. The differences between groups are evaluated using the independent samples t-test. Categorical variables are expressed as frequencies and proportions, and the differences between groups are evaluated using the chi-square test. A P-value less than 0.05 indicates statistical significance.

### 3.2 Comparison of baseline data between the two groups

In **Table 2**, compared with the normal sleep group, the sleep disorder group had a higher proportion of patients with type 2 diabetes (P = 0.039), hypertension (P = 0.030), coronary heart disease (P = 0.013), and stroke (P = 0.018). Additionally, the sleep disorder group showed significantly lower levels of body mass index, peripheral albumin, prealbumin, hemoglobin, and transferrin than the normal sleep group (all P < 0.001). However, there were no significant differences between the two groups in terms of demographic data and risk factors for NSCLC (all P > 0.05).

**Table 2:** Characteristics of the older patients at baseline.

Variable	Sleep disorder group	Normal sleep group	χ² or t value	P value
Total (n)	210 (100.0)	285 (100.0)	–	–
Demographic data				
Male (n)	109 (51.9)	132 (46.3)	1.512	0.219
Age (year)	72.1 ± 6.8	71.6 ± 6.2	0.843	0.399
Han nationality (n)	189 (90.0)	267 (93.7)	2.261	0.133
History of chronic disease				
Type 2 diabetes (n)	39 (18.6)	34 (11.9)	4.242	0.039
Hypertension (n)	100 (47.6)	108 (37.9)	4.693	0.030
Coronary heart disease (n)	64 (30.5)	59 (20.7)	6.186	0.013
Stroke (n)	31 (14.8)	23 (8.1)	5.571	0.018
Risk factors for NSCLC				
Cigarette smoking (n)	107 (51.0)	124 (43.5)	2.692	0.101
Occupational exposure (n)	37 (17.6)	38 (13.3)	1.727	0.189
Air pollution (n)	153 (72.9)	189 (66.3)	2.423	0.120
Ionizing radiation (n)	26 (12.4)	25 (8.8)	1.704	0.192
Familial inheritance (n)	56 (26.7)	62 (21.8)	1.607	0.205
Chronic lung diseases (n)	109 (51.9)	128 (44.9)	2.369	0.124
Nutritional data				
Body mass index (kg/m^2^)	20.0 ± 1.7	22.0 ± 2.2	11.302	<0.001
Peripheral albumin (g/L)	33.0 ± 1.7	35.7 ± 2.4	14.203	<0.001
Peripheral prealbumin (mg/L)	186.1 ± 18.9	213.9 ± 23.4	14.175	<0.001
Peripheral hemoglobin (g/L)	105.5 ± 7.4	120.2 ± 11.3	16.322	<0.001
Peripheral transferrin (g/L)	2.4 ± 0.2	2.6 ± 0.2	12.277	<0.001

NSCLC = Non-small cell lung cancer. Continuous variables are expressed as mean ± standard deviation. The differences between groups are evaluated using the independent samples t-test. Categorical variables are expressed as frequencies and proportions, and the differences between groups are evaluated using the chi-square test. A P-value less than 0.05 indicates statistical significance.

In **Table 3**, compared with the normal sleep group, the sleep disorder group had significantly higher levels of peripheral neutrophil count, monocyte count, CD8+ T cell proportion, C-reactive protein, and erythrocyte sedimentation rate (all P < 0.001). In contrast, the sleep disorder group showed significantly lower levels of peripheral lymphocyte count, CD3+ T cell proportion, CD4+ T cell proportion, immunoglobulin G, immunoglobulin A, immunoglobulin M, Complement C3, and Complement C4 (all P < 0.001).

**Table 3:** Peripheral immune and inflammatory status of the older patients at baseline.

Variable	Sleep disorder group	Normal sleep group	χ² or t value	P value
Total (n)	210 (100.0)	285 (100.0)	–	–
Leukocyte count				
Neutrophil (×10^-9^/L)	5.4 ± 0.8	4.5 ± 0.8	12.962	<0.001
Lymphocyte (×10^-9^/L)	1.4 ± 0.2	1.8 ± 0.4	14.893	<0.001
Monocyte (×10^-9^/L)	0.8 ± 0.1	0.6 ± 0.2	14.169	<0.001
Subsets of T lymphocytes				
CD3+ T cells (%)	47.1 ± 3.5	54.1 ± 5.9	15.310	<0.001
CD4+ T cells (%)	27.6 ± 3.0	31.1 ± 4.3	9.977	<0.001
CD8+ T cells (%)	32.5 ± 4.6	29.3 ± 4.7	7.388	<0.001
Immunoglobulin				
Immunoglobulin G (g/L)	8.4 ± 1.5	9.4 ± 1.5	7.421	<0.001
Immunoglobulin A (g/L)	1.7 ± 0.3	2.0 ± 0.3	11.689	<0.001
Immunoglobulin M (g/L)	0.7 ± 0.2	0.9 ± 0.2	11.509	<0.001
Complement				
Complement C3 (g/L)	0.9 ± 0.1	1.0 ± 0.1	11.204	<0.001
Complement C4 (g/L)	0.1 ± 0.1	0.2 ± 0.1	12.085	<0.001
Other				
C-reactive protein (mg/L)	18.8 ± 5.2	13.0 ± 5.1	12.607	<0.001
ESR (mm/h)	42.2 ± 9.3	28.6 ± 10.5	14.943	<0.001

ESR = Erythrocyte sedimentation rate. Continuous variables are expressed as mean ± standard deviation. The differences between groups are evaluated using the independent samples t-test. Categorical variables are expressed as frequencies and proportions, and the differences between groups are evaluated using the chi-square test. A P-value less than 0.05 indicates statistical significance.

In **Table 4**, compared with the normal sleep group, the sleep disorder group had a significantly higher proportion of patients with poorly differentiated tumor differentiation (P < 0.001), tumor stage IV (P = 0.032), and EGFR gene mutation (P = 0.037), as well as significantly elevated levels of peripheral carcinoembryonic antigen (P < 0.001), cytokeratin 19 fragment (P < 0.001), neuron-specific enolase (P < 0.001), lactate dehydrogenase (P < 0.001), and tumor mutation burden (P < 0.001). No significant differences were observed in other characteristics of NSCLC between the two groups (all P > 0.05).

**Table 4:** Characteristics of non-small cell lung cancer in the older patients at baseline.

Variable	Sleep disorder group	Normal sleep group	χ² or t value	P value
Total (n)	210 (100.0)	285 (100.0)	–	–
Histological type				
Adenocarcinoma (n)	128 (61.0)	153 (53.7)	2.603	0.107
Squamous cell carcinoma (n)	59 (28.1)	93 (32.6)	1.169	0.280
Large cell carcinoma (n)	23 (11.0)	39 (13.7)	0.824	0.364
Differentiation degree				
Well-Moderately (n)	76 (36.2)	150 (52.6)	13.173	<0.001
Poorly-Undifferentiated (n)	134 (63.8)	135 (47.4)		
Tumor stage				
IIIB/IIIC (n)	54 (25.7)	99 (34.7)	4.609	0.032
IV (n)	156 (74.3)	186 (65.3)		
Gene detection				
EGFR gene mutation (n)	41 (19.5)	36 (12.6)	4.372	0.037
ALK fusion gene (n)	8 (3.8)	17 (6.0)	1.171	0.279
ROS1 fusion gene (n)	4 (1.9)	12 (4.2)	2.055	0.152
BRAF gene mutation (n)	11 (5.2)	7 (2.5)	2.670	0.102
PD-L1 expression≥1% (n)	29 (13.8)	56 (19.6)	2.899	0.089
Peripheral tumor marker				
CEA (μg/L)	8.4 ± 2.0	7.2 ± 1.7	7.115	<0.001
CYFRA21-1 (ng/mL)	5.3 ± 1.2	4.5 ± 1.1	7.352	<0.001
NSE (ng/mL)	3.8 ± 0.9	3.1 ± 0.9	8.894	<0.001
Other peripheral marker				
LDH (U/L)	282.1 ± 44.7	256.8 ± 46.5	6.062	<0.001
TMB (Mut/Mb)	5.5 ± 1.5	4.4 ± 1.5	7.914	<0.001

EGFR = Epidermal growth factor receptor, ALK = Anaplastic lymphoma kinase, ROS1 = Proto-oncogene 1, receptor tyrosine kinase, BRAF = B-raf proto-oncogene, serine/threonine kinase, PD-L1 = Programmed death-ligand 1, CEA = Carcinoembryonic antigen, CYFRA21-1 = Cytokeratin 19 fragment, NSE = Neuron-specific enolase, LDH = Lactate dehydrogenase, TMB = Tumor mutation burden. Continuous variables are expressed as mean ± standard deviation. The differences between groups are evaluated using the independent samples t-test. Categorical variables are expressed as frequencies and proportions, and the differences between groups are evaluated using the chi-square test. A P-value less than 0.05 indicates statistical significance.

### 3.3 Comparison of follow-up data between the two groups

In **Table 5**, compared with the normal sleep group, the sleep disorder group had a significantly lower proportion of patients receiving ICI monotherapy (P = 0.021), a significantly higher proportion of patients with an ICI course of 3 to 6 months (P = 0.001), and a significantly lower proportion of patients with an ICI course of more than 12 months (P = 0.007).

**Table 5:** Treatment for the older patients during the 24-month follow-up.

Variable	Sleep disorder group	Normal sleep group	χ² or t value	P value
Total (n)	210 (100.0)	285 (100.0)	–	–
Treatment regimen				
ICI monotherapy (n)	77 (36.7)	134 (47.0)	5.297	0.021
ICI + CT (n)	105 (50.0)	127 (44.6)	1.436	0.231
ICI + RT (n)	24 (11.4)	20 (7.0)	2.905	0.088
ICI + CT + RT (n)	4 (1.9)	4 (1.4)	0.191	0.662
ICI type				
Pembrolizumab (n)	127 (60.5)	159 (55.8)	1.089	0.297
Nivolumab (n)	83 (39.5)	126 (44.2)		
ICI course				
3 to 6 months (n)	84 (40.0)	73 (25.6)	11.554	0.001
6 to 12 months (n)	78 (37.1)	115 (40.4)	0.523	0.470
More than 12 months (n)	48 (22.9)	97 (34.0)	7.294	0.007
ICI discontinuation reason				
PD (n)	76 (36.2)	92 (32.3)	0.824	0.364
Intolerable ADR (n)	32 (15.2)	36 (12.6)	0.693	0.405
Others (n)	102 (48.6)	157 (55.1)	2.058	0.151
CT regimen				
Pemetrexed + Cisplatin (n)	41 (19.5)	60 (21.1)	0.174	0.677
Paclitaxel + Carboplatin (n)	38 (18.1)	39 (13.7)	1.791	0.181
Gemcitabine + Cisplatin (n)	19 (9.0)	22 (7.7)	0.281	0.596
Others	11 (5.2)	10 (3.5)	0.890	0.345
RT dose				
≤50 Gy (n)	20 (9.5)	17 (6.0)	2.214	0.137
>50 Gy (n)	8 (3.8)	7 (2.5)	0.754	0.385
RT target				
Confined to the thorax (n)	22 (10.5)	21 (7.4)	1.472	0.225
Others (n)	6 (2.9)	3 (1.1)	2.206	0.138

ICI = Immune checkpoint inhibitor, CT = Chemotherapy, RT = Radiotherapy, CR = Complete response, PR = Partial response, SD = Stable disease, PD = Progressive disease, ADR = Adverse reaction. Continuous variables are expressed as mean ± standard deviation. The differences between groups are evaluated using the independent samples t-test. Categorical variables are expressed as frequencies and proportions, and the differences between groups are evaluated using the chi-square test. A P-value less than 0.05 indicates statistical significance.

In **Table 6**, compared with the normal sleep group, the sleep disorder group showed a significantly lower proportion of patients with CR/PR/SD as the ICI efficacy at 3 months (P = 0.002), a significantly lower number of surviving patients at 24 months (P = 0.003), and significantly shorter PFS and OS at 24 months (P = 0.003, P = 0.002, respectively). In contrast, the sleep disorder group had significantly higher proportions of patients experiencing gastrointestinal toxicity (P = 0.012), liver toxicity (P = 0.009), and lung toxicity (P = 0.026).

**Table 6:** Outcomes of the older patients during the 24-month follow-up.

Variable	Sleep disorder group	Normal sleep group	χ² or t value	P value
Total (n)	210 (100.0)	285 (100.0)	–	–
ICI efficacy at 3 months				
CR/PR/SD (n)	115 (54.8)	195 (68.4)	9.638	0.002
PD (n)	95 (45.2)	90 (31.6)		
ADR during the 24 months				
Skin toxicity (n)	15 (7.1)	12 (4.2)	2.016	0.156
Gastrointestinal toxicity (n)	23 (11.0)	14 (4.9)	6.378	0.012
Liver toxicity (n)	18 (8.6)	9 (3.2)	6.871	0.009
Endocrine toxicity (n)	12 (5.7)	7 (2.5)	3.477	0.062
Lung toxicity (n)	10 (4.8)	4 (1.4)	4.962	0.026
Outcome at 24 months				
Survival (n)	74 (35.2)	138 (48.4)	8.582	0.003
Death (n)	136 (64.8)	147 (51.6)		
Survival time at 24 months				
PFS (month)	10.8 ± 6.9	12.6 ± 6.6	2.949	0.003
OS (month)	16.2 ± 7.4	18.2 ± 6.8	3.172	0.002

ICI = Immune checkpoint inhibitor, CR = Complete response, PR = Partial response, SD = Stable disease, PD = Progressive disease, ADR = Adverse reaction, PFS = Progression-free survival, OS = Overall survival. Continuous variables are expressed as mean ± standard deviation. The differences between groups are evaluated using the independent samples t-test. Categorical variables are expressed as frequencies and proportions, and the differences between groups are evaluated using the chi-square test. A P-value less than 0.05 indicates statistical significance.

### 3.4 Multivariate regression analysis and Kaplan-Meier survival analysis

In **Table 7**, multivariate logistic regression analysis revealed that sleep disorders were associated with a reduced likelihood of achieving CR/PR/SD for ICI efficacy at 3 months (P = 0.002), an increased risk of gastrointestinal toxicity (P = 0.014), liver toxicity (P = 0.012), and lung toxicity (P = 0.036) during the 24-month follow-up, as well as a decreased probability of survival at 24 months (P = 0.004).

**Table 7:** Multivariate analysis for the association of sleep disorder with the outcome of the older patients during the 24-month follow-up.

Variable	Statistical method	P value	OR/HR	95%CI
ICI Efficacy at 3 months				
CR/PR/SD (Overall)	Multivariate logistic regression	0.002	0.559	0.386 ~ 0.808
CR/PR/SD (PD-L1≥1% subgroup)	Multivariate logistic regression	0.186	0.643	0.335 ~ 1.235
CR/PR/SD (PD-L1<1% subgroup)	Multivariate logistic regression	0.001	0.497	0.328 ~ 0.752
CR/PR/SD (Interaction: Sleep disorder×PD-L1)	Multivariate logistic regression	0.273	1.528	0.689 ~ 3.381
ADR during the 24 months				
Skin toxicity	Multivariate logistic regression	0.160	1.750	0.801 ~ 3.821
Gastrointestinal toxicity	Multivariate logistic regression	0.014	2.381	1.194 ~ 4.747
Liver toxicity	Multivariate logistic regression	0.012	2.875	1.265 ~ 6.535
Endocrine toxicity	Multivariate logistic regression	0.070	2.407	0.931 ~ 6.222
Lung toxicity	Multivariate logistic regression	0.036	3.512	1.086 ~ 11.358
Outcome and survival time at 24 months				
Survival	Multivariate logistic regression	0.004	0.580	0.402 ~ 0.836
PFS	Multivariate COX regression	0.048	0.834	0.696 ~ 0.999
OS	Multivariate COX regression	0.002	0.690	0.547 ~ 0.872

ICI = Immune checkpoint inhibitor, OR = Odds ratio, HR = Hazard ratio, 95%CI = 95% confidence interval, CR = Complete response, PR = Partial response, SD = Stable disease, ADR = Adverse reaction, PFS = Progression-free survival, OS = Overall survival. The multivariate model was adjusted for demographic data, disease history, risk factors for NSCLC, nutritional data, peripheral immune and inflammatory data, tumor characteristics, treatment regimen, and ICI course. A P-value less than 0.05 indicates statistical significance.

Further subgroup analysis based on programmed death-ligand 1 (PD-L1) expression level (≥1% vs. <1%) showed that the negative association between sleep disorders and ICI efficacy (CR/PR/SD) was more pronounced in the subgroup with PD-L1 expression < 1% (P = 0.001), while in the PD-L1 expression ≥1% subgroup, the association exhibited a similar trend but did not reach statistical significance (P = 0.186). The interaction test for “sleep disorder×PD-L1 expression” indicated no significant modifying effect of PD-L1 expression on the association between sleep disorders and ICI efficacy (P = 0.273).

Multivariate Cox regression analysis showed that sleep disorders were significantly associated with reduced PFS and OS during the 24 months (P = 0.048, P = 0.002, respectively).

In **Supplementary Figure 1**, Kaplan-Meier survival analysis indicated that sleep disorders were associated with decreased PFS and OS during the 24 months (P = 0.033, P = 0.002, respectively).

### 3.5 Supplementary analysis based on propensity score matching

Owing to baseline data imbalance, propensity score matching was used to adjust for baseline variables with statistical significance. After matching, 98 patients were included in each group.

**Supplementary Table 2** showed no significant differences in history of diseases, nutritional data, peripheral immune and inflammatory data, or NSCLC characteristics between the two groups (all P>0.05).

**Supplementary Table 3** indicated that the sleep disorder group had a lower proportion of CR/PR/SD for ICI efficacy at 3 months (P = 0.013), a higher incidence of adverse reaction at 24 months (P = 0.005), a lower survival rate at 24 months (P = 0.020), and shorter PFS and OS (P = 0.006, P = 0.018, respectively).

Multivariate logistic regression in **Supplementary Table 4** demonstrated that sleep disorders were associated with reduced CR/PR/SD at 3 months (P = 0.014), increased adverse reaction at 24 months (P = 0.006), and decreased survival probability at 24 months (P = 0.021).

Notably, due to reduced sample size, different types of adverse reaction were combined, and multivariate Cox regression was not performed; however, the available data were sufficient to support the aforementioned results.

### 3.6 Sample size and statistical power

Sample size and statistical power analysis were performed using G*Power 3.1.9.7 software. Taking the key results of the association between sleep disorders and OS from **Table 7** as an example, with a two-tailed test, a significance level of α=0.05, a test power of 1−β=0.95, and an effect size of ∣ρ∣=0.3, the minimum sample size required to achieve the ideal test power was calculated to be 134 cases. In this study, a total of 495 cases were actually enrolled, which was larger than this minimum sample size, indicating that the sample size met the requirements and could provide reliable statistical power support for the analysis of the association between sleep disorders and OS.

## 4 Discussion

The clinical significance of this study lies in providing potential reference for optimizing individualized ICI treatment regimens in older adults with NSCLC based on sleep disorder assessment. It also offers a new direction for exploring strategies to reduce the risk of treatment-related adverse events and improve patients’ quality of life as well as survival outcomes.

As is well-known, NSCLC has a high incidence in the older population, and older NSCLC patients constitute an important group for ICI treatment. Meanwhile, the incidence of sleep disorders is inherently high among the older population, and this issue becomes even more prominent when compounded by the combined effects of tumor condition, treatment-related side effects, and financial stress (18). Previous preliminary studies have suggested a potential association between sleep disorders and ICI efficacy (8–10). Therefore, in the key population of older NSCLC patients, in-depth exploration of the relationship between sleep disorders and the efficacy of ICI treatment holds significant clinical and research significance.

This study found that sleep disorders were associated with the efficacy of ICI treatment in older patients with NSCLC. Specifically, sleep disorders were associated with worse ICI efficacy (based on thoracic CT assessment results) at the 3-month follow-up, while being linked to a lower survival rate at the 24-month follow-up. Additionally, sleep disorders correlated with shorter PFS and OS over the 24-month period. These findings can corroborate each other, enhancing the credibility of the results. This study also confirmed that sleep disorders were significantly associated with the increased risk of adverse reactions across multiple systems (including the gastrointestinal tract, liver, and lungs) in older patients with NSCLC receiving ICI treatment. This finding indicates that sleep disorders are associated with both reduced treatment efficacy and compromised treatment safety, reflecting a dual negative correlation with treatment benefits and patient prognosis.

Notably, to address baseline data imbalance, this study first adjusted for potential confounding factors using multivariate logistic and Cox regression, followed by supplementary analysis with propensity score matching to calibrate baseline discrepancies. The consistency between the two sets of results further enhances the credibility of the conclusions.

PD-L1 is a core predictive biomarker for the efficacy of ICI therapy, and the impact of its expression level on treatment response has been widely validated. Subgroup analysis in this study revealed certain differences in the association between sleep disorders and ICI efficacy across the two PD-L1 expression subgroups. More importantly, however, the “sleep disorder × PD-L1” interaction test did not reach statistical significance. This result indicates that PD-L1 expression did not significantly modulate the association between sleep disorders and ICI efficacy.

In this study, the ICIs administered to patients were Pembrolizumab or Nivolumab, both of which are programmed death-1 (PD-1) inhibitors. These agents restore T-cell immunosurveillance function by blocking the PD-1/PD-L1 pathway (19, 20). From a mechanistic perspective, Nivolumab exhibits higher affinity for PD-1, while Pembrolizumab retains an intact Fc region, thus conferring relatively higher affinity for Fcγ receptors. Current evidence indicates that there is no significant difference in efficacy between the two agents in the treatment of NSCLC. Additionally, there was no statistical difference in drug distribution among different study groups, suggesting that this factor did not interfere with the primary study results.

It should be noted that a considerable proportion of patients in this study received concurrent chemotherapy and/or radiotherapy while undergoing ICI treatment, and there was a certain imbalance in the distribution of ICI monotherapy versus combination therapy between the two groups. However, this distribution difference has been adjusted for through multivariate analysis. Furthermore, all radiotherapy in this study was palliative in nature, and chemotherapy regimens used were the well-tolerated ones commonly applied in clinical practice for older NSCLC patients. Therefore, the adjustment via statistical methods and the relative homogeneity of treatment strategies ensure that including patients receiving ICI monotherapy and those on combination therapy in a unified analysis does not compromise the reliability of the results.

In addition, a small subset of patients with EGFR/ALK gene mutations received ICI-containing treatment regimens, which was consistent with the recommendations of clinical guidelines for advanced NSCLC during the study enrollment period. At that time, although tyrosine kinase inhibitors (TKIs) were the preferred first-line therapy for this patient population, the guidelines did not completely exclude the applicability of ICIs in specific clinical scenarios (e.g., poor baseline liver or kidney function, concurrent high PD-L1 expression, or low EGFR mutation allele frequency). Additionally, drug accessibility was an important factor taken into consideration by the medical team at that time.

In the present study, the PSQI was used to assess patients’ sleep status four times within the first 3 months of follow-up, and patients with significant sleep-disturbing factors during the 24-month follow-up were excluded to capture sleep status more comprehensively and accurately. However, it should be noted that sleep disorders may not be an independent pre-existing predictor of prognosis, but are more likely to be co-induced by tumor-related symptoms, ICI toxicities, and other factors, which is consistent with the actual clinical course of patients. Once such sleep disorders occur, they can still inversely affect ICI efficacy through pathways such as immune regulation and inflammatory response, forming an interactive relationship rather than a unidirectional causal one. Therefore, this does not diminish the clinical significance of the present study.

The relationship between sleep disorders and ICI efficacy reported in this study can be explained by multiple mechanisms. First, long-term sleep disorders disrupt the normal activation and proliferation of T cells (21, 22). Specifically, they reduce the number of effector T cells with anti-tumor properties and inhibit their cytotoxic function. Since the efficacy of ICIs depends on the body’s immune recognition and attack of tumor cells, this impairment of T cell function directly weakens the ability of ICIs to activate anti-tumor immunity. Second, sleep disturbances trigger or exacerbate systemic chronic inflammation, leading to increased release of pro-inflammatory cytokines such as interleukin-6 and tumor necrosis factor-α (23–25). These cytokines not only inhibit the activity of immune cells but also may promote the accumulation of immunosuppressive cells (e.g., regulatory T cells) in the tumor microenvironment, further impeding the anti-tumor immune response mediated by ICIs. Additionally, persistent sleep disorders affect patients’ nutrient absorption and metabolic balance, resulting in reduced muscle mass and physical fitness (26, 27). This lowers the body’s tolerance to treatment, which may indirectly impact patients’ adherence to therapy or interfere with the normal metabolic process of drugs in the body, ultimately exerting a negative effect on the efficacy of ICI treatment.

Notably, in the multivariate analysis of this study, a series of indicators related to immune dysfunction, inflammatory imbalance, and decreased physical reserve capacity were adjusted for; however, these measures did not offset the association between sleep disorders and ICI efficacy. This study suggests that this result does not negate the potential role of immune status, inflammatory levels, and physical reserve capacity in the aforementioned mechanisms. The reason is that immune-inflammatory responses and biological processes related to the body’s reserve capacity are highly complex, and there may be mutual regulatory interactions between these processes. The current indicator adjustments cannot fully cover their complex associations.

This study has certain limitations. First, although the sample size meets the requirements for basic statistical power, its scale remains limited and cannot support further subgroup analyses. This prevented the study from thoroughly investigating efficacy differences among patients receiving different ICI treatment regimens (e.g., monotherapy vs. combination therapy) or with varying degrees of sleep disorders (e.g., mild/moderate/severe). Second, since genes such as STK11, KEAP1, and KRAS were not routine clinical monitoring items at the time of patient enrollment in this study, relevant test data are lacking, and thus these genes could not be included in the analysis to explore whether these key driver genes might influence the association between sleep disorders and ICI efficacy. Finally, this study only verified the association between sleep disorders and the efficacy of ICI therapy in NSCLC, without in-depth exploration of the specific underlying mechanisms, and therefore failed to reveal the core biological pathways underlying the association between sleep disorders and treatment efficacy. Future studies can build on improved detection of the aforementioned genes, combined with larger sample sizes and stratified designs, to further clarify the population-specificity of the association between sleep disorders and ICI efficacy, and simultaneously conduct in-depth exploration of potential regulatory mechanisms, thereby providing more robust evidence for the development of targeted intervention strategies.

In conclusion, this study demonstrates that among older NSCLC patients receiving ICI treatment, sleep disorders are significantly associated with poorer treatment efficacy and a higher risk of multisystem adverse reactions, and this association remains valid after adjusting for various potential confounding factors. These results provide a preliminary clinical basis for optimizing individualized ICI treatment regimens and improving patient prognosis in older adults with NSCLC based on sleep assessment. They also suggest that future studies need to further explore the exact impact of sleep disorders on ICI efficacy and related mechanisms, laying a foundation for the development of targeted intervention strategies.

## Data Availability

The raw data supporting the conclusions of this article will be made available by the authors, without undue reservation.

## Supplementary material

Supplementary Figure 1 Kaplan-Meier survival analysis for the association of sleep disorder with the survival time at 24 months. **(A)** indicates the association of sleep disorder with the progression-free survival (P = 0.033); Supplemental Figure 1B indicates the association of sleep disorder with the overall survival (P = 0.002).

Supplementary Table 1 Definitions of risk factors for non-small cell lung cancer.

Supplementary Table 2 Characteristics of the older patients at baseline after propensity score matching.

Supplementary Table 3 Outcomes of the older patients during the 24-month follow-up after propensity score matching.

Supplementary Table 4 Multivariate analysis for the association of sleep disorder with the outcome during the 24-month follow-up after propensity score matching.
