# Supplementary material for: Relationship between sleep disorders and the efficacy of immune checkpoint inhibitors in older patients with non-small cell lung cancer
Source: Front Oncol. 2026 Jan 9;15:1713801. doi: 10.3389/fonc.2025.1713801 (PMC12827081; doi:10.3389/fonc.2025.1713801)
Supplement: Supplementary Figure 1 — Kaplan-Meier survival analysis for the association of sleep disorder with the survival time at 24 months. (A) indicates the association of sleep disorder with the progression-free survival (P = 0.033); Supplemental Figure 1B indicates the association of sleep disorder with the overall survival (P = 0.002). [file DataSheet1.docx]

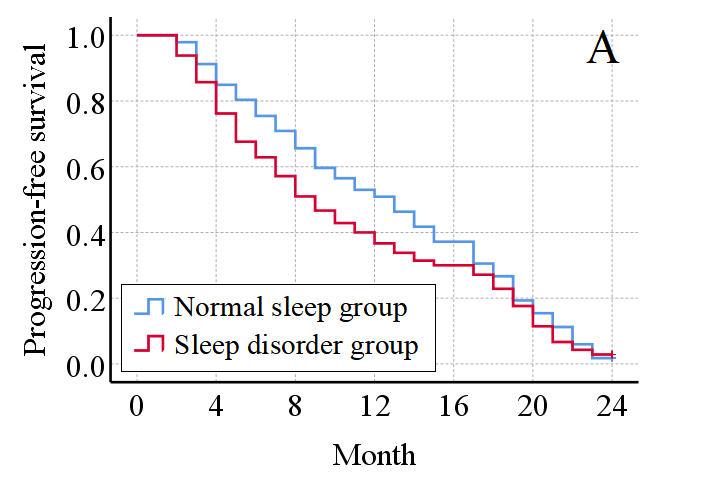


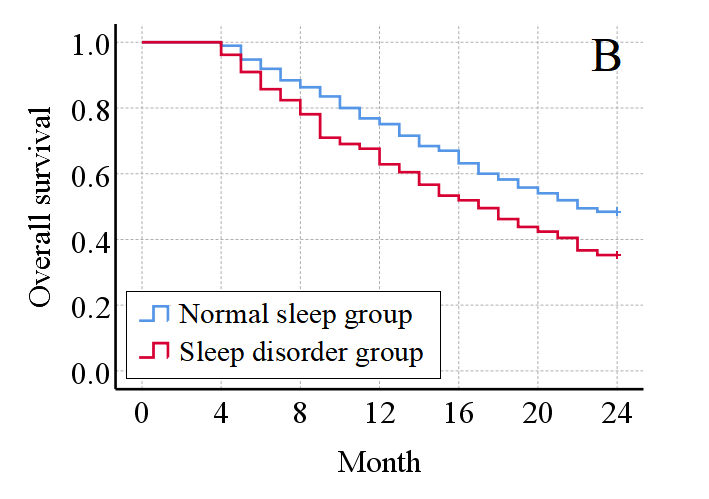


**Supplemental Figure 1 Kaplan-Meier survival analysis for the association of sleep disorder with the survival time at 24 months.**

Supplemental Figure 1A indicates the association of sleep disorder with the progression-free survival (P = 0.033); Supplemental Figure 1B indicates the association of sleep disorder with the overall survival (P = 0.002).

**Supplemental Table 1 Definitions of risk factors for non-small cell lung cancer**

| **Risk factor for NSCLC** | **Definition** |
| --- | --- |
| Cigarette smoking | Smoking at least 1 cigarette per day for more than 10 years. |
| Occupational exposure | Engaging in jobs related to asbestos or heavy metals for more than 4 hours per day (half a working day), lasting for more than 5 years. |
| Air pollution | Being exposed to vehicle exhaust, cooking fumes, etc. for more than 4 hours per day (half a working day), lasting for more than 10 years. |
| Ionizing radiation | Engaging in jobs related to radiation for more than 4 hours per day (half a working day), lasting for more than 5 years. |
| Familial inheritance | Having a family history of lung cancer, with immediate blood relatives or collateral relatives within three generations diagnosed with lung cancer. |
| Chronic lung diseases | Suffering from chronic lung diseases such as chronic bronchitis or chronic obstructive pulmonary disease for more than 10 years, or having pulmonary fibrosis for more than 5 years. |

Note: NSCLC = Non-small cell lung cancer.

**Supplemental Table 2 Characteristics of the older patients at baseline after propensity score matching**

| **Variable** | **Sleep disorder group** | **Normal sleep group** | **χ² or**  **t value** | **P value** |
| --- | --- | --- | --- | --- |
| Total (n) | 98 (100.0) | 98 (100.0) | － | － |
| Type 2 diabetes (n) | 16 (16.3) | 13 (13.3) | 0.364 | 0.546 |
| Hypertension (n) | 42 (42.9) | 39 (39.8) | 0.189 | 0.663 |
| Coronary heart disease (n) | 26 (26.5) | 22 (22.4) | 0.441 | 0.506 |
| Stroke (n) | 14 (14.3) | 10 (10.2) | 0.760 | 0.383 |
| Body mass index (kg/m^2^) | 20.7±1.8 | 21.0±2.1 | 0.982 | 0.374 |
| Peripheral albumin (g/L) | 34.1±1.6 | 34.4±2.3 | 1.001 | 0.318 |
| Peripheral prealbumin (mg/L) | 198.9±18.9 | 201.5±22.0 | 0.866 | 0.387 |
| Peripheral hemoglobin (g/L) | 111.4±7.1 | 113.2±10.5 | 1.403 | 0.162 |
| Peripheral transferrin (g/L) | 2.4±0.2 | 2.5±0.3 | 1.176 | 0.241 |
| Neutrophil (×10^-9^/L) | 5.1±0.8 | 5.0±0.9 | 1.378 | 0.170 |
| Lymphocyte (×10^-9^/L) | 1.5±0.2 | 1.6±0.4 | 0.953 | 0.342 |
| Monocyte (×10^-9^/L) | 0.8±0.2 | 0.7±0.2 | 1.635 | 0.104 |
| CD3+ T cells (%) | 50.1±3.6 | 50.7±6.1 | 0.819 | 0.414 |
| CD4+ T cells (%) | 28.9±3.1 | 29.3±4.0 | 0.855 | 0.393 |
| CD8+ T cells (%) | 31.2±4.9 | 30.7±5.1 | 0.758 | 0.449 |
| Immunoglobulin G (g/L) | 8.9±1.5 | 9.0±1.4 | 0.852 | 0.396 |
| Immunoglobulin A (g/L) | 1.8±0.3 | 1.9±0.3 | 0.919 | 0.359 |
| Immunoglobulin M (g/L) | 0.7±0.2 | 0.8±0.2 | 0.745 | 0.457 |
| Complement C3 (g/L) | 0.9±0.1 | 1.0±0.2 | 0.790 | 0.430 |
| Complement C4 (g/L) | 0.2±0.1 | 0.1±0.1 | 0.534 | 0.594 |
| C-reactive protein (mg/L) | 16.5±5.4 | 16.2±5.5 | 0.456 | 0.649 |
| ESR (mm/h) | 36.7±9.7 | 35.2±10.6 | 0.999 | 0.319 |
| Poorly-Undifferentiated (n) | 58 (59.2) | 51 (52.0) | 1.013 | 0.314 |
| Tumor stage IV (n) | 69 (70.4) | 65 (66.3) | 0.377 | 0.539 |
| EGFR gene mutation (n) | 17 (17.3) | 14 (14.3) | 0.345 | 0.557 |
| PD-L1 expression≥1% (%) | 16.7±2.2 | 17.0±2.6 | 0.891 | 0.374 |
| CEA (μg/L) | 8.0±2.0 | 7.7±1.8 | 1.137 | 0.257 |
| CYFRA21-1 (ng/mL) | 5.1±1.2 | 4.9±1.1 | 1.069 | 0.286 |
| NSE (ng/mL) | 3.6±0.9 | 3.5±0.9 | 0.705 | 0.481 |
| LDH (U/L) | 276.2±44.4 | 271.1±46.9 | 0.778 | 0.437 |
| TMB (Mut/Mb) | 5.2±1.6 | 4.9±1.6 | 1.516 | 0.131 |

Note: ESR = Erythrocyte sedimentation rate, EGFR = Epidermal growth factor receptor, PD-L1 = Programmed death-ligand 1, CEA = Carcinoembryonic antigen, CYFRA21-1 = Cytokeratin 19 fragment, NSE = Neuron-specific enolase, LDH = Lactate dehydrogenase, TMB = Tumor mutation burden. A P-value less than 0.05 indicates statistical significance.

**Supplemental Table 3 Outcomes of the older patients during the 24-month follow-up after propensity score matching**

| **Variable** | **Sleep disorder group** | **Normal sleep group** | **χ² or**  **t value** | **P value** |
| --- | --- | --- | --- | --- |
| Total (n) | 98 (100.0) | 98 (100.0) | － | － |
| **ICI efficacy at 3 months** |  |  |  |  |
| CR / PR / SD (n) | 51 (52.0) | 68 (69.4) | 6.182 | 0.013 |
| PD (n) | 47 (48.0) | 30 (30.6) |  |  |
| **ADR during the 24 months** |  |  |  |  |
| Present (n) | 33 (33.7) | 16 (16.3) | 7.864 | 0.005 |
| Absent (n) | 65 (66.3) | 82 (83.7) |  |  |
| **Outcome at 24 months** |  |  |  |  |
| Survival (n) | 32 (32.7) | 48 (49.0) | 5.407 | 0.020 |
| Death (n) | 66 (67.3) | 50 (51.0) |  |  |
| **Survival time at 24 months** |  |  |  |  |
| PFS (month) | 10.4±6.4 | 13.0±6.9 | 2.780 | 0.006 |
| OS (month) | 16.1±7.1 | 18.5±7.0 | 2.392 | 0.018 |

Note: ICI = Immune checkpoint inhibitor, CR = Complete response, PR = Partial response, SD = Stable disease, PD = Progressive disease, ADR = Adverse reaction, PFS = Progression-free survival, OS = Overall survival. A P-value less than 0.05 indicates statistical significance.

**Supplemental Table 4 Multivariate analysis for the association of sleep disorder with the outcome during the 24-month follow-up after propensity score matching**

| **Variable** | **Multivariate logistic regression** | | |
| --- | --- | --- | --- |
|  | **P value** | **OR** | **95%CI** |
| **ICI efficacy at 3 months** |  |  |  |
| CR / PR / SD | 0.014 | 0.479 | 0.267 ~ 0.859 |
| **ADR during the 24 months** |  |  |  |
| Present | 0.006 | 2.602 | 1.318 ~ 5.136 |
| **Outcome at 24 months** |  |  |  |
| Survival | 0.021 | 0.505 | 0.283 ~ 0.901 |

Note: ICI = Immune checkpoint inhibitor, OR = Odds ratio, 95%CI = 95% confidence interval, CR = Complete response, PR = Partial response, SD = Stable disease, ADR = Adverse reaction, PFS = Progression-free survival, OS = Overall survival. The multivariate model was adjusted for demographic data, disease history, risk factors for NSCLC, nutritional data, peripheral immune and inflammatory data, tumor characteristics, treatment regimen, and ICI course. A P-value less than 0.05 indicates statistical significance.
